# Functions and mechanisms of circular RNAs in cancer stem cells and therapy resistance

**DOI:** 10.3389/fcell.2026.1809429

**Published:** 2026-04-01

**Authors:** Yixiao Yuan, Dahang Zhang, Juan Wang, Xiulin Jiang, Lincan Duan

**Affiliations:** 1 The Third Affiliated Hospital of Kunming Medical University, Peking University Cancer Hospital Yunnan, Kunming, Yunan, China; 2 College of Life Science, University of Chinese Academy of Sciences, Beijing, China; 3 Department of Thoracic Surgery, Pu’er People’s Hospital, Pu’er, Yunan, China

**Keywords:** biomarkers, cancer stem cells, circRNA, CSC markers, drug resistance, epigenetic regulation, solid tumors, therapeutic strategies

## Abstract

Cancer stem cells (CSCs) play a central role in tumor initiation, progression, recurrence, and therapy resistance. Their abilities for self-renewal, multi-lineage differentiation, and strong resistance make conventional chemotherapy, targeted therapy, and radiotherapy insufficient to completely eradicate tumors. In recent years, circular RNAs (circRNAs), a class of novel non-coding RNAs, have been shown to regulate CSC properties through multiple mechanisms, including acting as miRNA sponges, interacting with proteins, modulating signaling pathways, and encoding small peptides. Accumulating evidence indicates that circRNAs are aberrantly expressed in CSCs across various tumor types, including liver cancer, lung cancer, breast cancer, gastric cancer, prostate cancer, ovarian cancer, glioma, and acute myeloid leukemia, influencing stemness and drug sensitivity via specific signaling pathways or regulatory networks. CircRNAs have potential as biomarkers for diagnosis, prognosis, and therapy resistance prediction, as well as promising therapeutic targets. Strategies targeting oncogenic circRNAs, such as siRNA or shRNA delivered via liposomes, can effectively suppress CSC stemness and resistance and may be combined with chemotherapy, targeted therapy, or immunotherapy. Despite challenges such as incomplete mechanistic understanding, CSC heterogeneity, and limited clinical validation, advances in single-cell sequencing, circRNA interference, and nanocarrier delivery provide new opportunities for clinical translation. Overall, circRNAs play critical roles in maintaining CSC stemness, modulating drug resistance, and promoting tumor progression, offering novel avenues for overcoming therapy-resistant CSCs and for early diagnosis, prognosis assessment, and personalized treatment.

## 1 Introduction

CSCs are a small subpopulation of tumor cells with stem cell-like properties. They were first identified in hematological malignancies and later confirmed in various solid tumors (Rao et al., 2022). The core features of CSCs include self-renewal, multi-lineage differentiation, and pronounced drug resistance (Toh et al., 2017). These characteristics make CSCs major contributors to tumor recurrence, metastasis, and therapeutic failure. For instance, in breast cancer, liver cancer, and glioma, CSCs can survive conventional chemotherapy or radiotherapy and reinitiate tumor growth, leading to disease relapse or aggressive metastasis (Saw et al., 2024). Clinically, therapy resistance remains a leading cause of cancer-related mortality. CSCs achieve drug resistance through multiple mechanisms, including quiescence to evade cell cycle-dependent drugs, drug efflux mediated by ABC transporters, enhanced DNA repair, upregulation of anti-apoptotic signaling, and microenvironment-mediated protection (Amiri-Farsani et al., 2024). These traits limit the effectiveness of conventional chemotherapy and targeted therapy in eliminating CSCs, highlighting the urgent need to identify novel regulatory targets.

In recent years, epigenetic regulation has emerged as a key mechanism in maintaining CSC stemness and therapy resistance (Miyoshi et al., 2019). Beyond classical DNA methylation, histone modifications, and non-coding RNA regulation, circular RNAs (circRNAs) have attracted increasing attention as a novel epigenetic regulator (Toh et al., 2017). CircRNAs are covalently closed RNA molecules generated by back-splicing, which confer high stability and resistance to exonuclease-mediated degradation, resulting in long intracellular half-lives (Patop and Kadener, 2018). They regulate gene expression through diverse mechanisms, such as acting as miRNA sponges, interacting with RNA-binding proteins (RBPs), modulating transcription and splicing, and, in some cases, being translated into proteins or peptides (Patop and Kadener, 2018). Accumulating evidence indicates that circRNAs play critical roles in regulating CSC self-renewal, plasticity, and drug resistance.

Although a recent comprehensive review has summarized the general functions and mechanisms of circRNAs in cancer stem cells (CSCs) (Jain et al., 2026), our work provides a more focused perspective. As a mini-review, we specifically summarize studies from the past 5 years regarding the regulatory roles and mechanisms of circRNAs in CSCs across different cancer types. Importantly, compared with previous reviews that mainly emphasize CSC self-renewal and stemness maintenance, we place particular emphasis on the mechanisms by which circRNAs regulate CSC-mediated therapeutic resistance. In addition, we further discuss the potential of circRNAs as therapeutic targets and their clinical applications related to CSCs, while also addressing the current limitations and challenges associated with targeting circRNAs. Therefore, although previously provides a broader overview of circRNAs in CSC biology (Jain et al., 2026), our review complements existing literature by highlighting the emerging role of circRNAs in CSC-associated therapy resistance and their translational potential. By analyzing current research on circRNA regulation of CSCs, we hope to provide a theoretical foundation for targeting CSCs to overcome therapy resistance and to inspire the development of novel anti-resistance therapeutic strategies.

## 2 Functions and mechanisms of circRNAs

**FIGURE 1:**
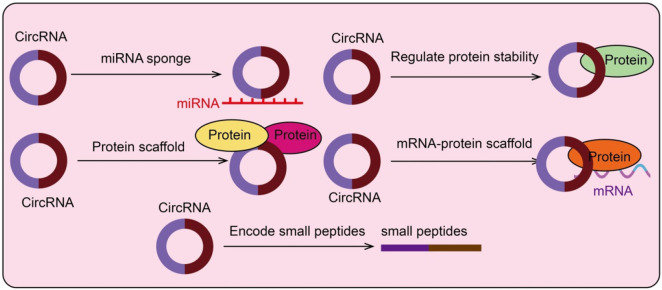
Functional mechanisms of circRNAs in cancer stem cells. CircRNAs can act as miRNA sponges, protein scaffolds, mRNA-protein scaffolds, regulate protein stability, or encode small peptides.

CircRNAs exert diverse regulatory functions and can influence cell fate and tumor behavior through multiple mechanisms, playing key roles in the self-renewal and drug resistance of CSCs. Figure 1 depicts the key mechanisms through which circRNAs regulate cancer stem cells, including miRNA sponging, protein or mRNA-protein scaffolding, modulation of protein stability, and peptide coding (Figure 1).

### 2.1 Acting as miRNA sponges

CircRNAs can bind specific miRNAs via complementary binding sites, reducing their inhibitory effects on target genes and thereby indirectly upregulating gene expression (Tao et al., 2021). For example, in some cancer stem cells, certain circRNAs act as sponges for the miR-524–4p or miR-9-3p families, enhancing the expression of stemness transcription factors such as SOX2, OCT4, and NANOG (Yang et al., 2020). This mechanism not only maintains CSC stemness but also promotes chemoresistance (Yang et al., 2020).

### 2.2 Serving as protein scaffolds

Some circRNAs can simultaneously bind multiple proteins to form complexes, facilitating protein–protein interactions and signal transduction (Zhou et al., 2020). For instance, circRNAs can function as scaffolds for proteins involved in Notch, β-catenin, or PI3K/AKT signaling pathways, enhancing signal activity and sustaining CSC self-renewal and drug resistance (Zhou et al., 2020).

### 2.3 Regulating protein stability

CircRNAs can modulate protein stability by directly binding proteins or interfering with their degradation pathways (Wesselhoeft et al., 2018). Certain circRNAs interact with E3 ubiquitin ligases or protein degradation complexes, preventing the degradation of stemness transcription factors or anti-apoptotic proteins such as BCL-2 and β-catenin, thereby promoting CSC survival and drug resistance.

### 2.4 Acting as mRNA-Protein scaffolds

CircRNAs can simultaneously bind specific mRNAs and RNA-binding proteins to form ternary complexes, enhancing mRNA stability or translation efficiency (Yi et al., 2024). For example, in liver cancer stem cells, circRNAs stabilize complexes of mRNAs and their binding proteins, increasing the expression of drug-resistance-related proteins and thereby enhancing chemoresistance (Yuan et al., 2025). For example, our recent study shows that Circ0515 can act as a molecular scaffold to recruit RBM45, maintaining the stability of SDHB transcripts, thereby promoting mitochondrial succinate metabolism and enhancing cisplatin resistance in lung adenocarcinoma (Yuan et al., 2025).

### 2.5 Encoding small peptides

Although most circRNAs are non-coding, a few contain internal open reading frames (ORFs) and can be translated into small peptides (Yi et al., 2024). These peptides participate in signaling regulation and can affect CSC stemness or drug-resistance pathways. For instance, circRNA-derived peptides can modulate Wnt/β-catenin or PI3K/AKT signaling, promoting CSC self-renewal and chemoresistance (Yi et al., 2024).

## 3 Overview of cancer stem cells

### 3.1 Definition and characteristics of CSCs

CSCs are a subpopulation of tumor cells with stem cell-like properties, including self-renewal and multi-lineage differentiation (Miyoshi et al., 2019). CSCs not only sustain tumor growth and heterogeneity but also exhibit significant resistance to chemotherapy, targeted therapy, and radiotherapy, making them key drivers of tumor recurrence and metastasis (Miyoshi et al., 2019). CSCs have been identified in various hematological malignancies and solid tumors, including acute myeloid leukemia (AML), breast cancer, liver cancer, lung cancer, gastric cancer, prostate cancer, ovarian cancer, and glioma (Miyoshi et al., 2019). While the proportion, characteristics, and microenvironmental dependencies of CSCs vary among tumor types, their core functions—maintaining stemness and enhancing drug resistance—are highly conserved.

### 3.2 CSC markers

CSCs are commonly identified by surface or nuclear markers. In solid tumors, widely studied markers include:Surface markers: CD44, CD24, CD133, EpCAM. Stemness transcription factors: SOX2, OCT4, NANOG. These markers not only serve as identifiers for CSCs but also actively regulate stemness, proliferation, and therapy resistance (Peixoto and Lima, 2018). For example, CD44 and EpCAM are closely associated with tumor migration and drug resistance, whereas SOX2 and OCT4 maintain self-renewal and activate anti-apoptotic signaling (Peixoto and Lima, 2018).

### 3.3 CSC-mediated therapy resistance

CSCs play a central role in therapy resistance through multiple mechanisms. They are often quiescent or slowly proliferating, allowing them to evade cell cycle-dependent chemotherapeutic drugs (Steinbichler et al., 2018). High expression of ATP-binding cassette (ABC) transporters, such as ABCB1 and ABCG2, actively pumps drugs out of cells, reducing intracellular drug concentrations (Steinbichler et al., 2018). CSCs also enhance anti-apoptotic signaling (e.g., BCL-2 family proteins) and DNA repair capacity, enabling survival under chemotherapy- or radiotherapy-induced stress (Steinbichler et al., 2018).

#### 3.3.1 Chemotherapy resistance

CSCs are a major source of chemoresistance. Their quiescent state reduces sensitivity to cell cycle-dependent agents such as cisplatin, paclitaxel, and doxorubicin. High ABC transporter activity, upregulated anti-apoptotic signals, and efficient DNA repair collectively allow CSCs to survive chemotherapy and reinitiate tumor growth, leading to relapse (Najafi et al., 2019).

#### 3.3.2 Targeted therapy resistance

CSCs also contribute to resistance against targeted therapies. In EGFR-driven non-small cell lung cancer (NSCLC), CSCs activate downstream EGFR signaling (PI3K/AKT, MAPK) and upregulate stemness transcription factors (SOX2, OCT4), conferring resistance to EGFR tyrosine kinase inhibitors (EGFR-TKIs). In hepatocellular carcinoma, CSCs resist sorafenib through enhanced stemness pathways (Wnt/β-catenin, Notch, Hedgehog) and microenvironment-mediated protection (Najafi et al., 2019). This resistance arises from CSCs’ intrinsic signaling networks and adaptive heterogeneity.

#### 3.3.3 Radiotherapy resistance

CSCs generally display high radioresistance due to multiple mechanisms. They efficiently repair DNA double-strand breaks via homologous recombination and non-homologous end joining (Has et al., 2021). Their enhanced antioxidant capacity reduces reactive oxygen species (ROS)-induced DNA damage. Microenvironmental niches, such as perivascular regions or bone marrow, provide additional survival signals. Upregulation of anti-apoptotic proteins, including BCL-2 and Survivin, further contributes to radioresistance (Has et al., 2021). These features allow CSCs to survive radiotherapy and drive tumor recurrence and invasion.

## 4 circRNAs in CSC biology and drug resistance

**FIGURE 2:**
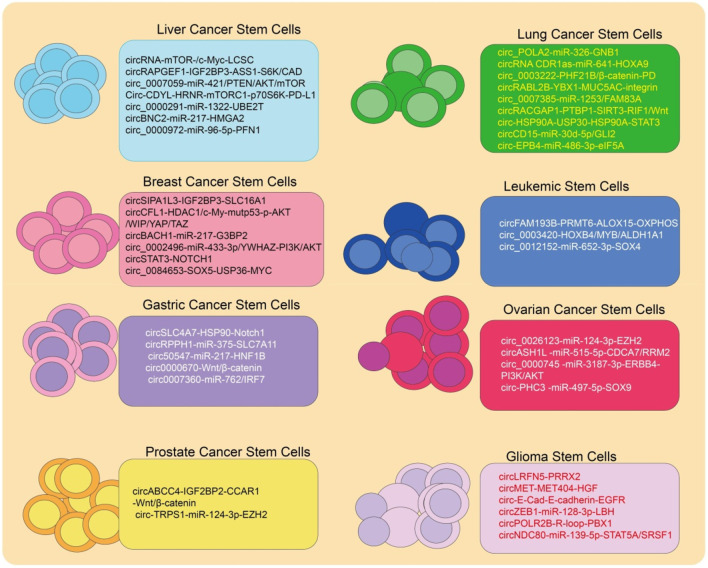
CircRNAs identified in various CSCs. Different colors represent CSCs from different cancer types, and listed circRNAs are implicated in their regulation.

Figure 2 highlights circRNAs identified in different cancer stem cells, with colors representing cancer types and listing circRNAs involved in their regulation.

### 4.1 circRNAs in liver cancer stem cells (LiCSCs) and drug resistance

LiCSCs possess high self-renewal capacity, tumor-initiating potential, and notable phenotypic plasticity, representing a critical source of hepatocellular carcinoma (HCC) recurrence and metastasis (Chang et al., 2019). Common markers include EpCAM, CD133, CD90, CD44, OV6, and ALDH1. LiCSCs typically maintain stemness through activation of Wnt/β-catenin, Notch, Hedgehog, and PI3K/AKT signaling pathways (Gu et al., 2020). Regarding drug resistance, LiCSCs are closely associated with resistance to sorafenib and lenvatinib, mediated by mechanisms such as upregulation of ABC transporters, enhanced anti-apoptotic signaling, activation of EMT, and metabolic reprogramming (Gu et al., 2020). LiCSCs play key roles in HCC progression, stemness maintenance, and resistance to chemotherapy and targeted therapies. Multiple studies have demonstrated that circRNAs regulate LiCSCs stemness and drug sensitivity through diverse mechanisms. CircRNA-mTOR promotes nuclear translocation of the RNA-binding protein PSIP1 and activates the PSIP1/c-Myc pathway, enhancing LiCSCs stemness, driving HCC progression, and mediating lenvatinib resistance (Tang Y. et al., 2025); circRAPGEF1, stabilized by METTL3-mediated m6A modification, sponges IGF2BP3, disrupts ASS1 mRNA, and activates the S6K/CAD signaling pathway, increasing stemness and decreasing sorafenib sensitivity (Shi et al., 2025); circ_0007059 inhibits stemness, proliferation, and migration via the miR-421/PTEN/AKT/mTOR pathway (Hui et al., 2022); Circ-CDYL stabilizes HRNR and activates mTORC1-p70S6K signaling, enhancing stemness and PD-L1 expression while reducing sensitivity to anti-PD-L1 immunotherapy (Fu et al., 2025); circ_0000291 promotes stemness and anti-apoptotic capacity through sponging miR-1322 to upregulate UBE2T (Wang et al., 2022); circBNC2 enhances stemness, glycolysis, and tumor progression by sponging miR-217 to upregulate HMGA2 (Feng et al., 2023); circ_0000972 sponges miR-96–5p to regulate PFN1, inhibiting stemness, sphere formation, and EMT, and its overexpression suppresses LiCSCs growth (Tang J. et al., 2025). Overall, these circRNAs significantly regulate LiCSCs stemness and drug resistance via miRNA sponging, m6A modification, protein interactions, and signaling pathways. They serve as molecular markers for LiCSCs function and prognosis and provide potential targets for reversing drug resistance and improving HCC therapy, offering new strategies for targeted therapy and immunotherapy.

### 4.2 circRNAs in lung cancer stem cells (LuCSCs) and drug resistance

Lung cancer stem cells, particularly in non-small cell lung cancer (NSCLC), exhibit tumorigenic potential and pronounced resistance to radiotherapy, chemotherapy, and targeted therapy (Rowbotham et al., 2022). Common markers include CD133, ALDH1, CD44, EpCAM, and SOX2, often accompanied by aberrations in driver genes such as EGFR and KRAS (Rowbotham et al., 2022). Resistance mechanisms include EGFR-TKI resistance, radioresistance, and cisplatin resistance, primarily mediated by enhanced DNA damage repair, increased ROS detoxification, and activation of Notch/Wnt signaling. LuCSCs play crucial roles in lung cancer progression, stemness maintenance, and resistance to chemotherapy and immunotherapy. Studies have shown that multiple circRNAs regulate LuCSCs stemness and drug sensitivity through diverse mechanisms (Rowbotham et al., 2022). Circ_POLA2 promotes stemness and sphere formation by sponging miR-326 to upregulate GNB1 and is associated with poor prognosis (Fan et al., 2020); circRNA CDR1as enhances NSCLC stemness and cisplatin resistance via sponging miR-641 to upregulate HOXA9, and its knockdown can reverse resistance (Zhao et al., 2020); hsa_circ_0003222 inhibits stemness and anti-PD-L1 immune resistance through the PHF21B/β-catenin pathway (Li et al., 2021); circRABL2B suppresses stemness and increases erlotinib sensitivity by inhibiting MUC5AC and downregulating the integrin β4/pSrc/p53 pathway (Lu et al., 2023); circ_0007385 regulates stemness and proliferation via the miR-1253/FAM83A axis, and its exosomal form promotes LuCSCs stemness and drug resistance (Ning et al., 2022); circRACGAP1 enhances stemness and metastasis by recruiting PTBP1 to stabilize SIRT3 and activating RIF1/Wnt/β-catenin signaling (Xiong et al., 2023); circ-HSP90A stabilizes HSP90A via USP30 and activates STAT3 while regulating immune evasion through the miR-424–5p/PD-L1 axis (Lei et al., 2023); circCD151 and circ_0044516 regulate stemness, proliferation, and invasion through miR-30d-5p/GLI2 and miR-136/MAT2A axes, respectively (Zhao et al., 2021); circ-EPB41 enhances stemness marker expression and self-renewal via sponging miR-486–3p to regulate eIF5A (Jin et al., 2022). Collectively, these circRNAs significantly influence LuCSCs stemness and drug resistance via miRNA sponging, protein interactions, m6A modification, and signaling pathways, serving as markers for LuCSCs function and prognosis and offering potential targets to overcome resistance and improve therapy.

### 4.3 circRNAs in breast cancer stem cells (BCSCs) and drug resistance

Breast cancer stem cells display strong invasiveness and metastatic potential, particularly in triple-negative breast cancer (TNBC) (Zhang et al., 2023). Classical phenotypes include CD44^+^CD24^-^/low and ALDH1^+^. Active signaling pathways in BCSCs include Wnt, Notch, Hedgehog, and TGF-β. BCSCs are closely associated with resistance to paclitaxel, doxorubicin, trastuzumab, and endocrine therapy, involving mechanisms such as enhanced EMT, upregulation of stemness transcription factors, and HER2/PI3K signaling abnormalities (Zhang et al., 2023). CircRNAs play crucial roles in BCSC stemness maintenance, metabolic regulation, and drug resistance. CircSIPA1L3, via EIF4A3-mediated circularization and cytoplasmic transport, stabilizes IGF2BP3 and regulates SLC16A1 and RAB11A mRNA or sponges miR-665, promoting glycolysis and lactate secretion, thereby recruiting tumor-promoting macrophages and accelerating breast cancer progression (Liang et al., 2024). CircCFL1 acts as a scaffold to enhance HDAC1/c-Myc interactions, stabilizing c-Myc, upregulating mutp53, and activating the p-AKT/WIP/YAP/TAZ pathway, promoting stemness, proliferation, and immune evasion in TNBC (Wang et al., 2024a). CircBACH1 sponges miR-217 to upregulate G3BP2, enhancing stemness, migration, and paclitaxel resistance (Xia et al., 2023); circ_0002496 activates PI3K/AKT signaling via the miR-433–3p/YWHAZ axis, promoting stemness and tumor growth (Yang et al., 2023); circSTAT3 and circMIB1 regulate stemness, migration, and chemoresistance via exosomal transfer or peptide translation, targeting NOTCH1 signaling or blocking RNF213-mediated ubiquitination (Ye et al., 2025; Yang et al., 2025); circ_0084653, induced by SOX5 and stabilized by USP36-mediated MYC deubiquitination, sponges miR-1323 to release SOX5 expression, further maintaining stemness and tumor progression (Dong et al., 2024). Overall, these circRNAs enhance BCSC stemness and drug resistance through regulation of miRNAs, RNA-binding proteins, signaling pathways, and metabolic reprogramming, while also influencing immune evasion and the tumor microenvironment, serving as functional markers and potential targets for diagnosis, prognosis, and therapeutic intervention.

### 4.4 circRNAs in gastric cancer stem cells (GCSCs) and drug resistance

Gastric cancer stem cells participate in tumor initiation and recurrence (Rao et al., 2022). Common markers include CD44, LGR5, CD133, and ALDH1. Stemness maintenance relies on Wnt/β-catenin and Hedgehog signaling (Rao et al., 2022). GCSCs are associated with resistance to 5-FU, cisplatin, and taxanes, mediated by enhanced DNA repair, EMT induction, and upregulation of anti-apoptotic proteins such as Bcl-2 (Rao et al., 2022). CircRNAs regulate GCSC stemness, proliferation, migration, invasion, and drug resistance. CircSLC4A7, predominantly nuclear, binds HSP90 to activate Notch1 signaling, enhancing stemness and pro-tumor behavior (Hui et al., 2023); circRPPH1 sponges miR-375 to upregulate SLC7A11, inhibiting ferroptosis and promoting stemness (Liu et al., 2023); circ50547 sponges miR-217 to upregulate HNF1B, promoting stemness, proliferation, and drug resistance, with diagnostic potential in serum exosomes (Zang et al., 2024); tobacco exposure induces circ0000670 in GCSC-derived exosomes, activating stemness gene expression, EMT, and Wnt/β-catenin signaling, promoting gastric tumorigenesis (Liang et al., 2023); circ0007360 inhibits stemness, migration, and proliferation through the miR-762/IRF7 axis, showing potential tumor-suppressive activity (Xing et al., 2022). Overall, circRNAs maintain GCSC stemness and contribute to proliferation, invasion, drug resistance, and environmental response, representing potential diagnostic, prognostic, and therapeutic targets.

### 4.5 circRNAs in prostate cancer stem cells (PCSCs) and drug resistance

Prostate cancer stem cells exhibit high plasticity, particularly in castration-resistant prostate cancer (CRPC) (Adamowicz et al., 2017). Common markers include CD44, CD133, α2β1 integrin, and ALDH1 (Adamowicz et al., 2017). PCSCs are associated with resistance to androgen deprivation therapy (ADT) and enzalutamide, involving AR signaling reprogramming, upregulation of stemness transcription factor SOX2, and neuroendocrine differentiation (Adamowicz et al., 2017). CircRNAs regulate PCSC stemness, proliferation, migration, and invasion. CircABCC4 is highly expressed in PCa tissues and cells and correlates with poor overall survival; METTL3-mediated m6A modification upregulates circABCC4, which recruits IGF2BP2 to stabilize CCAR1 mRNA, activating Wnt/β-catenin signaling and promoting stemness and metastasis (Huang et al., 2023). Knockdown of circABCC4 or METTL3 suppresses PCSC stemness, migration, invasion, and tumor growth (Huang et al., 2023). Circ-TRPS1 is upregulated in high-grade PCa tissues and cells, associated with invasive phenotypes, and promotes stemness and proliferation by sponging miR-124–3p to upregulate EZH2; inhibition of miR-124–3p or EZH2 overexpression reverses circ-TRPS1 knockdown effects. CircABCC4 and circ-TRPS1 maintain PCSC stemness and pro-tumor traits via miRNA sponging, RNA-binding proteins, and key signaling pathways, representing potential therapeutic targets.

### 4.6 circRNAs in ovarian cancer stem cells (OCSCs) and drug resistance

Ovarian cancer stem cells are closely linked to peritoneal dissemination and recurrence (Ma et al., 2023). Common markers include CD44, CD117, ALDH1, and EpCAM, with active Notch and Wnt signaling pathways (Ma et al., 2023). OCSCs typically exhibit cisplatin and paclitaxel resistance, mediated by enhanced DNA repair, upregulation of anti-apoptotic proteins, and tumor microenvironment support (Ma et al., 2023). CircRNAs regulate OCSC stemness, proliferation, migration, and chemoresistance. Hsa_circ_0026123 is highly expressed in ovarian cancer tissues and cells and promotes stemness and tumor growth by sponging miR-124–3p to upregulate EZH2; knockdown inhibits stemness and cancer progression (Yang et al., 2021). CircASH1L is upregulated in cisplatin-resistant A2780/DDP and SKOV3/DDP cells, and its knockdown promotes apoptosis, ferroptosis, and reverses cisplatin resistance by sponging miR-515–5p to regulate the CDCA7/RRM2 axis (Feng et al., 2025); *in vivo* silencing enhances cisplatin sensitivity. Circ_0000745 sponges miR-3187–3p to upregulate ERBB4 and activate PI3K/AKT signaling, enhancing proliferation, invasion, EMT, and stemness, stabilized by IGF2BP2 (Wang et al., 2021); circ-PHC3 sponges miR-497–5p to upregulate SOX9, promoting stemness and tumorigenicity, with knockdown effects reversible by SOX9 overexpression or miR-497–5p inhibition (Wang H. et al., 2023). These circRNAs maintain OCSC stemness, drive tumor progression, and confer drug resistance through regulation of miRNAs, RNA-binding proteins, and signaling pathways, representing potential diagnostic markers and therapeutic targets.

### 4.7 circRNAs in glioma stem cells (GSCs) and drug resistance

Glioma stem cells are critical in glioblastoma (GBM) progression and recurrence, with markers including CD133, Nestin, SOX2, and OLIG2 (Agosti et al., 2024). GSCs exhibit strong resistance to radiotherapy and temozolomide (TMZ), mediated by enhanced DNA repair, active Notch and STAT3 signaling, low ROS levels, and vascular niche protection (Agosti et al., 2024). CircRNAs regulate GSC stemness and malignancy. CircLRFN5 induces ferroptosis by promoting PRRX2 degradation, suppressing proliferation, stemness, and neurosphere formation (Jiang et al., 2022a); circMET encodes MET404 protein, activating MET receptor independently of HGF to enhance self-renewal and invasion (Zhong et al., 2023); circ-E-Cad encodes secreted E-cadherin (C-E-Cad), activating EGFR signaling to maintain tumorigenicity (Gao et al., 2021); circZEB1 sponges miR-128–3p to upregulate LBH and forms a FUS/circZEB1/LBH positive feedback loop, promoting stemness (Zhang et al., 2024); circPOLR2B regulates POLR2B R-loop structures to upregulate PBX1, enhancing malignant phenotypes (Lin et al., 2024); circNDC80 sponges miR-139–5p to promote proliferation and stemness (Wang Y. et al., 2023); exosomal circCMTM3 is absorbed by differentiated glioma cells, modulating the STAT5A/SRSF1 feedback loop to promote angiogenic phenotypes (Wang C. et al., 2024). Other circRNAs, including circVPS8, circGNB1, circRPPH1, and circKPNB1, enhance proliferation, self-renewal, stemness, and tumorigenicity via ferroptosis regulation, RNA-binding proteins, and signaling pathways such as NF-κB, JAK2/STAT3, and TGF-β (Jiang et al., 2022b; Xu et al., 2022; Hu et al., 2023). These studies demonstrate the key role of circRNAs in GSC biology and provide potential diagnostic markers and therapeutic targets for GBM.

### 4.8 circRNAs in leukemic stem cells (LSCs) and drug resistance

Acute myeloid leukemia (AML) LSCs are a quiescent subpopulation with self-renewal and tumor-initiating capacity, representing the main cause of chemotherapy resistance and disease relapse (Long et al., 2022). LSCs rely on bone marrow microenvironment support, with common markers including CD34^+^CD38^−^, CD123, CD33, TIM-3, and CLL-1, with phenotypic variations among AML subtypes (Long et al., 2022). Stemness is maintained via Wnt/β-catenin, Notch, Hedgehog, PI3K/AKT, and BCL-2 signaling, with metabolism biased toward oxidative phosphorylation. Resistance mechanisms include quiescence-mediated chemotherapy evasion, ABC transporter-mediated drug efflux, enhanced DNA repair, anti-apoptotic protein upregulation, and microenvironment protection. CircRNAs regulate LSC stemness, drug tolerance, and redox state (Long et al., 2022). CircFAM193B downregulation interacts with PRMT6 to regulate ALOX15 transcription, enhancing mitochondrial OXPHOS and antioxidant capacity to resist chemotherapy-induced cell death (Yang et al., 2024); hsa-circ_0003420 downregulation is associated with LSC survival and the HOXB4/MYB/ALDH1A1 axis, while its overexpression inhibits stemness and induces apoptosis (Lin et al., 2021); circ_0012152 sponges miR-652–3p to upregulate SOX4, promoting proliferation and drug resistance, and its knockdown increases apoptosis and blocks cell cycle progression (Chen et al., 2024); circPVT1, enriched in AML-derived exosomes, modulates LSC-microenvironment signaling, promoting tumor growth and immune suppression (Wu et al., 2024); Bortezomib treatment under oxidative stress downregulates m6A regulator WTAP, altering expression of m6A-modified circRNAs such as circHIPK3, affecting protein folding and oxidative stress responses (Iaiza et al., 2024). Overall, these circRNAs regulate stemness markers, miRNA axes, redox balance, and exosome signaling, sustaining LSC survival, drug resistance, and AML progression, providing new molecular insights and potential therapeutic targets.

## 5 CircRNAs in clinical applications and potential value related to cancer stem cells (CSCs)

### 5.1 circRNAs as biomarkers

CircRNAs exhibit high stability, tissue specificity, and detectability in circulation across various cancer stem cells, making them promising clinical biomarkers. In diagnostics, specific circRNAs show differential expression in blood, exosomes, or tumor tissues, enabling early cancer detection. For example, exosomal circRNAs in gastric cancer and AML, such as circ50547 and circPVT1, are significantly elevated in patient serum, highlighting their potential for liquid biopsy application (Iaiza et al., 2024). Moreover, circRNAs can predict prognosis and therapeutic response. Studies indicate that highly expressed oncogenic circRNAs are often associated with enhanced CSC traits, tumor invasiveness, and drug resistance, suggesting their potential as indicators for patient survival and recurrence risk. In addition, circRNAs demonstrate unique advantages in predicting drug resistance; for instance, in AML, ovarian cancer, and glioma, circRNAs regulate stem cell-related resistance pathways, offering guidance for individualized therapeutic strategies.

### 5.2 CircRNAs as therapeutic targets

Given their critical roles in maintaining CSC stemness, promoting drug resistance, and driving tumor progression, circRNAs have emerged as potential therapeutic targets. Targeting oncogenic circRNAs or their regulatory networks, including miRNA axes, signaling pathways, and RNA-binding proteins, can inhibit CSC proliferation, stemness maintenance, and resistance. For example, interfering with circRNAs such as circABCC4, circRPPH1, and circPHC3, which are active in CSCs, can markedly reduce stem cell properties and invasive potential (Wang H. et al., 2023; Xu et al., 2022; Fan et al., 2021). CircRNA-targeting strategies can also be combined with chemotherapy, targeted therapy, or immunotherapy to enhance efficacy and overcome resistance. Advances in technologies such as CRISPR/Cas13, circRNA-specific RNA interference (circRNA-specific RNAi), and nanocarrier-based delivery may enable highly specific and efficient circRNA modulation, offering new avenues for anti-CSC therapies (Aquino-Jarquin, 2024). RNA interference (RNAi) provides a direct and specific means to target oncogenic circRNAs in CSCs. By designing siRNAs or shRNAs against circRNAs, expression of oncogenic circRNAs can be effectively suppressed, reducing CSC stemness, proliferation, and drug resistance. However, naked siRNAs are susceptible to nuclease degradation *in vivo*, are rapidly cleared, and may induce non-specific immune responses, making efficient and safe delivery systems essential. Liposomes, as classical nucleic acid carriers, have been widely applied in circRNA intervention studies due to their biocompatibility, ability to carry large RNA molecules, and modifiable targeting ligands. Liposomes can encapsulate siRNAs within lipid bilayers, enabling systemic or local administration, protecting against plasma degradation, and facilitating uptake into CSCs via membrane fusion or endocytosis. In CSC models, liposome-delivered siRNAs targeting oncogenic circRNAs such as circABCC4, circRPPH1, and circPHC3 effectively reduce CSC markers (e.g., CD44, SOX2, OCT4) and suppress tumor sphere formation, migration, and drug resistance (Wang et al., 2024b). Furthermore, surface modification of liposomes with antibodies or small-molecule targeting ligands enhances siRNA accumulation in CSCs, minimizing effects on normal tissues. Combining liposome-mediated circRNA siRNA delivery with chemotherapy or targeted therapy not only suppresses CSC activity alone but also improves therapeutic efficacy and overcomes resistance (Batlle and Clevers, 2017). For instance, in glioma or ovarian cancer models, circRNA-targeted liposome-siRNA combined with chemotherapy significantly reduces tumor burden and recurrence rates. With advances in nanotechnology and gene editing, liposome-mediated siRNA interference targeting circRNAs holds promise as a novel clinical strategy to specifically target CSCs (Batlle and Clevers, 2017).

### 5.3 Limitations and challenges of targeting circRNAs as therapeutic targets

Despite the promising potential of circRNAs as therapeutic targets in cancer, several limitations and challenges remain. First, the functional mechanisms of many circRNAs are still incompletely understood, particularly regarding their context-dependent roles in different tumor types and cellular states. Second, the efficient and specific delivery of circRNA-targeting strategies, such as RNA interference, antisense oligonucleotides, or CRISPR-based approaches, remains technically challenging *in vivo*. Off-target effects, limited tissue specificity, and potential immune responses may also restrict their clinical application. In addition, the high stability and complex biogenesis of circRNAs make their precise modulation more difficult compared with linear RNAs. Furthermore, the heterogeneity of cancer stem cells across tumors may influence the therapeutic efficacy of circRNA-targeting strategies. Therefore, further mechanistic studies and the development of more efficient delivery systems are required before circRNA-based therapies can be successfully translated into clinical applications.

## 6 Discussion and future perspectives

Although circRNAs exhibit broad potential in CSC research, several limitations remain. First, the mechanisms of circRNA function are not fully elucidated, particularly regarding their specific roles across different tumor types and CSC subpopulations. Second, the inherent heterogeneity of CSCs leads to complex resistance mechanisms, posing challenges for circRNA-targeted interventions. In addition, current studies predominantly rely on *in vitro* cell lines and animal models, with insufficient validation in clinical samples, limiting translational applications.

In addition to extensive preclinical studies, several ongoing clinical investigations are beginning to explore the translational potential of circRNAs as diagnostic or prognostic biomarkers in cancer. As summarized in previous reviews, several recent clinical studies investigating circRNAs have begun to emerge (Jain et al., 2026). For example, a clinical study (ClinicalTrials.gov ID: NCT06042842) is evaluating the clinical utility of plasma circRNA hsa_circ_0004001 as a non-invasive biomarker for the early diagnosis of hepatocellular carcinoma (HCC), with the aim of distinguishing malignant from non-malignant hepatic disorders and comparing its diagnostic performance with the conventional biomarker alpha-fetoprotein (AFP). Another study (ClinicalTrials.gov ID: NCT06617585) is investigating the role of circDENND4C in epithelial ovarian cancer to assess its potential relevance in early diagnosis and disease progression. In addition, the multicenter prospective study REBORN (ClinicalTrials.gov ID: NCT04464122) is examining circRNAs derived from tumor-educated platelets as novel biomarkers for the diagnosis and treatment response evaluation in pulmonary and gastro-entero-pancreatic neuroendocrine neoplasms. Furthermore, a clinical investigation in breast cancer patients (ClinicalTrials.gov ID: NCT05771337) is assessing the diagnostic and prognostic value of circulating circRNAs, including hsa_circ_0001785 (Circ-ELP3) and hsa_circ_100219 (Circ-FAF1), and their correlation with established tumor markers such as CEA and CA15-3. Collectively, these emerging clinical studies highlight the growing interest in circRNAs as potential biomarkers in cancer diagnosis and disease monitoring. However, large-scale validation studies and standardized detection approaches are still required before circRNA-based biomarkers can be widely implemented in clinical practice.

With technological advancements, circRNA research is poised for breakthroughs. Single-cell sequencing can delineate circRNA expression profiles and regulatory networks in different CSC subpopulations, providing a basis for precise targeting. CircRNA interference technologies and nanocarrier-based delivery systems enable highly specific *in vivo* modulation, offering safe and efficient intervention (Peiris-Pagès et al., 2016). Moreover, multimodal combination strategies, integrating circRNA targeting with chemotherapy or immunotherapy, may overcome drug resistance and improve clinical outcomes. Overall, circRNAs play crucial roles in maintaining CSC stemness, regulating drug resistance, and driving tumor progression, showing high potential for clinical translation. In the future, circRNAs may serve as novel targets for anti-resistant CSC therapies and offer new strategies for early cancer diagnosis, prognosis assessment, and personalized treatment. Further exploration of circRNA mechanisms and applications will facilitate the translation of CSC-related therapies from bench to bedside.
